# Mutational profiling of colorectal cancers with microsatellite instability

**DOI:** 10.18632/oncotarget.5997

**Published:** 2015-10-16

**Authors:** Elaine I. Lin, Li-Hui Tseng, Christopher D. Gocke, Stacy Reil, Dung T. Le, Nilofer S. Azad, James R. Eshleman

**Affiliations:** ^1^ Departments of Pathology, and the Sidney Kimmel Comprehensive Cancer Center, Johns Hopkins University School of Medicine, Baltimore, Maryland, USA; ^2^ Department of Medical Genetics, National Taiwan University Hospital, Taipei, Taiwan; ^3^ Departments of Oncology, and the Sidney Kimmel Comprehensive Cancer Center, Johns Hopkins University School of Medicine, Baltimore, Maryland, USA

**Keywords:** microsatellite instability, colorectal cancer, mutation profiling, mTOR pathway, PTEN

## Abstract

Microsatellite instability (MSI) is caused by defective mismatch repair in 15–20% of colorectal cancers (CRCs). Higher mutation loads in tumors with mismatch repair deficiency can predict response to pembrolizumab, an anti-programmed death 1 (PD-1) immune checkpoint inhibitor. We analyzed the mutations in 113 CRCs without MSI (MSS) and 29 CRCs with MSI-High (MSI-H) using the 50-gene AmpliSeq cancer panel. Overall, MSI-H CRCs showed significantly higher mutations than MSS CRCs, including insertion/deletion mutations at repeat regions. MSI-H CRCs showed higher incidences of mutations in the *BRAF*, *PIK3CA*, and *PTEN* genes as well as mutations in the receptor tyrosine kinase families. While the increased mutations in *BRAF* and *PTEN* in MSI-H CRCs are well accepted, we also support findings of mutations in the mTOR pathway and receptor tyrosine kinase family genes. MSS CRCs showed higher incidences of mutations in the *APC*, *KRAS* and *TP53* genes, confirming previous findings. NGS assays may be designed to detect driver mutations for targeted therapeutics and to identify tumors with high mutation loads for potential treatment with immune checkpoint blockade therapies. Further studies may be warranted to elucidate potential targeted therapeutics against mutations in the mTOR pathway and the receptor tyrosine kinase family in MSI-H CRCs as well as the benefit of anti-PD-1 immunotherapy in hypermutated MSS CRCs or other cancers.

## INTRODUCTION

Microsatellites (or short tandem repeats, STRs) are repetitive elements of 1–6 nucleotides tandemly repeated 10–60 times [1]. The repetitive nature of microsatellites makes them vulnerable to aberrant slippage during replication *in vivo* [2]. During replication *in vivo*, the mismatch repair (MMR) machinery normally repairs these mistakes to maintain the overall stability of microsatellites [3]. Human MMR genes (*MLH1*, *MSH2*, *MSH6* and *PMS2*) are homologues of those first identified in bacteria and yeast (*mutS* and *mutL*) [4, 5]. When the MMR machinery is defective, these slippage events go unrepaired and this results in microsatellite instability (MSI), defined as deviation in the number of tandem repeats in the tumor when compared to normal [6, 7, 8].

Microsatellite instability is observed in approximately 15–20% of colorectal cancers (CRCs) [9]. Of this, 2–3% is due to germline mutations of MMR genes (Lynch syndrome) and the majority is due to somatic changes, mostly hypermethylation in the *MLH1* promoter region. Mismatch repair defects can be detected by immunohistochemical studies to determine loss of MMR proteins or functionally, by testing microsatellite length at a panel of loci [10, 11, 12]. An aberrant number of repeats, as compared to the number of repeats in the germline genome, in 30% or more of the examined microsatellite loci is defined as microsatellite instability-high (MSI-H) [13]. CRCs with defective MMR have a mutation rate that is 100 to 1000 fold greater than that of CRCs with intact MMR [14]. These mutations occur in roughly equal numbers of frameshifts and base substitution mutations in the *HPRT* reporter gene, indicative of the mutations that occur in all genes and thereby creating mutational profiles distinct than those of microsatellite-stable (MSS) CRCs [15, 16, 17].

MSI-H CRCs often occur in the right colon and show mucinous and/or poorly differentiated histomorphology as well as profound lymphocyte infiltration [18]. In contrast with most solid tumors that have poorly differentiated histomorphology, MSI-H CRCs carry a superior outcome than MSS CRCs [7, 19]. A hypothesis has been proposed that hypermutation in MSI-H CRCs induces neoantigens and tumor infiltrating lymphocytes, a process that is attenuated by the strong expression of the immunosuppressive PD-1 receptor ligand (PD-L1) and several other immunosuppressive ligands [20, 21]. This hypothesis is supported by recent studies showing associations of the mutation burden or neoantigen burden with the responsiveness to immunotherapy [22, 23, 24, 25]. A superior progression-free survival was observed in MSI-H CRC patients, receiving pembrolizumab, an anti-Programmed Death-1 (PD-1) monoclonal antibody that blocks this pathway, thereby preventing apoptosis of tumor infiltrating lymphocytes [25]. Currently, MSI is a diagnostic marker for Lynch syndrome screening, a prognostic marker for better outcome in early stage disease, a negative predictor of 5-Fluorouracil (5-FU) response, [26] and a positive predictor for anti-PD-1 immunotherapy.

Massively parallel sequencing or next-generation sequencing (NGS) technology has revolutionized not only genome discovery, but also clinical molecular diagnostics. Molecular pathology is transitioning from conventional single gene assays, such as gold standard Sanger sequencing, towards a variety of NGS assays including small targeted gene panels for specific tumors, comprehensive mutational profiling of hundreds of cancer-related genes, and even whole exome sequencing [27]. NGS assays have shown both a high analytic sensitivity and a broad reportable range in the clinical diagnostic setting [28, 29]. In a previous retrospective analysis for quality assessment in a *Clinical Laboratory Improvement Amendments* (CLIA)-certified laboratory, we demonstrated the robust performance characteristics of an NGS assay using the AmpliSeq Cancer Hotspot Panel and Personal Genome Machine to detect a panel of *KRAS*, *NRAS*, *BRAF*, and *PIK3CA* genes (CRC panel) for prediction of anti-EGFR resistance [30, 31]. In this study, we analyzed the AmpliSeq Panel for 50 genes in 142 specimens submitted for both CRC panel and MSI testing in order to elucidate the mutational profile of MSI-H CRCs. Certain results of the AmpliSeq panel correlate with MSI-H tumors; however, no single criteria completely identifies these cancers.

## RESULTS

Of the 142 samples tested for both the AmpliSeq panel and MSI, 113 were MSS and 29 were MSI-H. There were no significant differences in age or gender between the MSS and MSI-H groups (Table 1). The MSS group contained a higher incidence of stage IV disease in the submitted samples while the MSI-H group showed a higher incidence of right-sided colorectal cancers. Metastatic tumors were examined in 29 of 113 MSS tumors submitted for testing, as compared to only 1 of 29 MSI-H tumors. The germline status of the patients with MSI CRCs is unknown.

**Table 1:** Demography of microsatellite stable (MSS) and microsatellite instability-high (MSI-high) tumors

	MSS (*n* = 113)	MSI-H (*n* = 29)	*P* value
Median age (range)	49 (25–84)	47 (26–89)	0.13
Gender			0.54
female	55 (49%)	16 (55%)	
male	58 (51%)	13 (45%)	
Location of primary tumors*			<0.01
right	35 (32%)	18 (64%)	
left	73 (68%)	10 (36%)	
Stage^†^			<0.01
I-III	53 (47%)	22 (76%)	
IV	59 (53%)	7 (24%)	
Specimens tested			<0.01
primary	84 (74%)	28 (97%)	
distant metastasis	29 (26%)	1 (3%)	

* Location of primary tumors was not known in 5 MSS tumors and 1 MSI-H tumor.

† Staging of tumors was not known on one MSS tumor.

### Higher mutation frequency in MSI-H tumors

**Figure 1:**
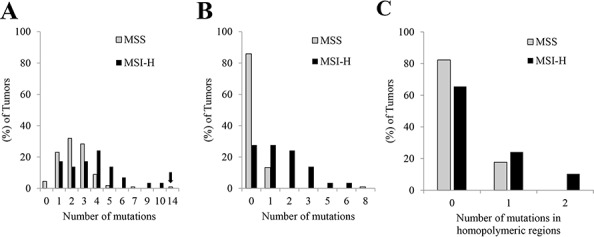
Histogram of the number of mutations detected by AmpliSeq panel in MSS tumors (*n* = 113) and MSI-H tumors (*n* = 29) **A.** Arrow indicates the outlier MSS tumor, CRC 131, with 14 mutations. **B.** Histogram of the number of uncommon mutations detected by AmpliSeq panel in MSS tumors (*n* = 113) and MSI-H tumors (*n* = 29). Commonly mutated genes in colorectal cancers *(APC, BRAF, CTNNB1, FBXW7, KRAS, NRAS, PIK3CA, SMAD4 and TP53)* were excluded. **C.** Histogram of the number of mutations in homopolymeric regions detected by AmpliSeq panel in MSS tumors (*n* = 113) and MSI-H tumors (*n* = 29)

There were 229 non-synonymous single nucleotide mutations (2.0 mutations per tumor), 33 deletion mutations (0.29 per tumor), 3 insertion/duplication mutations (0.026 per tumor), and 4 splicing mutations (0.035 per tumor) from the 113 MSS tumors compared to 92 non-synonymous single nucleotide mutations (3.2 mutations per tumor), 12 deletion mutations (0.41 per tumor), 3 insertion/duplication mutations (0.10 per tumor), and 1 splicing mutation (0.03 per tumor) from the 29 MSI-H tumors. MSI-H tumors showed a higher combined mutation frequency (3.7 ± 2.2 per tumor vs. 2.4 ± 1.6 in MSS tumors, mean ± 1 SD, *P* = < 0.001) (Figure 1A). In one MSS tumor metastasis, CRC131, 14 mutations were detected, substantially higher than the mean plus 3 standard deviations of either MSS tumors (7.2) or even MSI-high tumors (10.3). Ten mutations were detected in a second metastatic tumor from this patient. Only 5 of these were in common between the two metastases (i.e. 9 of them were unique to the first metastasis, and 5 of them unique to the second metastasis), suggesting a significant level of clonal evolution since the two lesions physically parted.

In MSS CRCs, the current AmpliSeq panel detected hotspot mutations in 8 of the top 10 genes most commonly mutated in CRC (*APC*, *TP53*, *KRAS*, *PIK3CA*, *FBXW7*, *SMAD4*, *NRAS* and *CTNNB1*). In MSI-H CRCs, the panel detected hotspot mutations in 2 of these 10 genes (*BRAF* and *APC*) [16, 17]. When mutations within the 9 genes listed previously were excluded, MSI-H tumors showed an 8-fold higher relative mutation frequency (1.6 ± 1.5 per tumor vs. 0.2 ± 0.8 in MSS tumors, mean ± 1 SD, *P* < 0.001) (Figure 1B). Thirteen MSI-H tumors showed more mutations than the mean plus 3 standard deviations of the MSS tumors (1.2, excluding specimen CRC131). Except for CRC 131, MSS tumors showed either 1 mutation or no mutation (0.1 ± 0.3, mean ± 1 SD).

### Mutational profiling of MSS tumors and MSI-H tumors

Mutations detected within the 9 top genes listed previously to be most commonly involved in CRCs were compared (Table 2, top). MSS tumors showed a higher incidence of *TP53* mutations (63% vs. 31%), *APC* mutations (47% vs. 34%), and *KRAS* mutations (46% vs. 31%) whereas MSI-H tumors showed significantly higher incidences of *BRAF* mutations (34% vs. 5%) and *PIK3CA* mutations (34% vs. 15%).

**Table 2:** Mutational profiling of MSS tumors and MSI-H tumors

Gene*	MSS (*n* = 113)	MSI-H (*n* = 29)	*P* value
*APC*	53 (47%)	10 (34%)	0.23
*BRAF*	6 (5%)^†^	10 (34%)	<0.001
*CTNNB1*	6 (5%)	1 (3%)	1
*FBXW7*	6 (5%)	4 (14%)	0.12
*KRAS*	52 (46%)	9 (31%)	0.15
*NRAS*	5 (4%)	3 (10%)	0.36
*PIK3CA*	17 (15%)	10 (34%)	0.02
*SMAD4*	12 (11%)	0 (0%)	0.13
*TP53*	71 (63%)	9 (31%)	<0.01
*AKT1*	1 (1%)	1 (3%)	0.30
*PTEN*	3 (3%)	8 (28%)	<0.001
mTOR^‡^	20 (18%)	17 (59%)	<0.001

* The 9 most commonly mutated genes in CRC are listed at the top.

† 4 p.V600E and 2 p.D594G mutations.

‡ mTOR pathway: mutation in *PIK3CA, PTEN* and/or *AKT1* genes.

In MSI-H tumors, the most common mutations observed outside of the 9 most common genes were located within the *PTEN* gene. Thirteen *PTEN* mutations were detected in 8 of the 29 (28%) MSI-H tumors as compared to only 3 of the 113 (3%) MSS tumors (*P* < 0.001). All 3 *PTEN* mutations in the MSS tumors were single base substitutions. On the other hand, among the 13 *PTEN* mutations in MSI-H tumors, there were 5 single base substitutions seen in 4 tumors and 8 indel variants seen in 6 tumors (Table 3). When only single base substitutions were analyzed, MSI-H tumors still showed a significantly higher mutation frequency of the *PTEN* gene (14% vs. 3%, *P* = 0.03). The mutation frequency within the mTOR pathway (*PIK3CA*, *PTEN* and/or *AKT1* genes) was significantly higher in the MSI-H tumors (55% vs. 18%, *P* < 0.001). Of the single MSI-H tumor with an *AKT1* mutation, *PIK3CA* and *PTEN* were not mutated. However, *PIK3CA* and *PTEN* mutations found in MSI-H tumors were not mutually exclusive of each other.

**Table 3:** *PTEN* mutations in colorectal cancers

Case	*PTEN* mutations^†^	Repeat region^‡^	Exon
MSS			
131	p.Y177Ter (c.531T > G)		6
133	p.R233Ter (c.697C > T)		7
181	p.A126T (c.376G > A)		5
MSI-H			
48	p.T319Ter (c.954_957del)	delTACT of (TACT)2	8
143	p.N323Mfs*21 (c.968delA)	delA of (A)6	8
218	p.L325R (c.974T > G)		8
218	p.K267Rfs*9 (c.800delA)	delA of (A)6	7
240	p.R234W (c.700C > T)		7
264	p.I122N (c.365T > A)		5
264	p.R130Q (c.359G > A)		
264	p.N323Mfs*21 (c.968delA)	delA of (A)6	8
273	p.S10I (c.29G > T)		1
MSIH22	p.Y178Ffs*6 (c.531_532dupTT)	dupTT	6
MSIH22	p.K267Rfs*9 (c.800delA)	delA of (A)6	7
MSIH24	p.K267Rfs*9 (c.800delA)	delA of (A)6	7
MSIH24	p.N323Mfs*21 (c.968delA)	delA of (A)6	8

† Frameshifts are indicated by “fs*” followed by the number of foreign amino acids potentially forming a neoepitope.

‡ Mutations involving homopolymer or short tandem repeat regions of the *PTEN* gene.

Similar trends were observed when only stage I-III primary tumors were analyzed. MSS tumors (*n* = 53) showed a significantly higher incidence of *TP53* mutations (60% vs. 32%, *P* = 0.02), while MSI-H tumors (*n* = 22) showed significantly higher incidences *BRAF* mutations (2% vs. 41%, *P* < 0.001) and *PTEN* mutations (4% vs. 32%, *P* < 0.01). Mutations within the mTOR pathway (*PIK3CA*, *PTEN* and/or *AKT1*) were observed in 10 (19%) MSS tumors and in 13 (59%) MSI-H tumors (*P* < 0.001).

### Mutations in the receptor tyrosine kinase (RTK) genes

The AmpliSeq panel detected hot spot mutations in class I (*EGFR*, *ERBB2* and *ERBB4*), class II (*CSF1R*, *FLT3*, *KIT* and *PDGFRA*), class IV (*FGFR1*, *FGFR2*, and *FGFR3*), class VI (*MET*), and class XIV (*RET*) RTK genes. Thirteen RTK mutations were detected in 9 of 29 (31%) MSI-H tumors as compared to only 6 RTK mutations detected in 2 of 113 (2%) MSS tumors (*P* < 0.001). Five of the 6 RTK mutations were seen in the single hypermutated MSS tumor, CRC131 (Table 4). Seven of 19 RTK mutations have been reported in the COSMIC database. Among the 12 mutations not reported in the COSMIC database, other types of mutations at the same amino acid position were reported in 8 of them.

**Table 4:** Mutations in the receptor tyrosine kinase (RTK) genes

Cases	RKT mutations, reported in COSMIC	RKT mutations, not reported in COSMIC
MSS		
131	ERBB4: S303Y (c.908C > A)	ERBB4: N365T (c.1094A > C) ERBB4: R232Ter (694C > T)*
		KIT: T500S (c.1498A > T)
		KIT: R634Q (c.1901G > A)*
266	KIT: V50M (c.148G > A)	
MSI-H		
61	ERBB2: L755S (c.2264T > C) KIT: R49C (c.145C > T)	
85	FGFR3: R640W (1918C > T)	FGFR3: G697S (2089G > A)*
252		EGFR: G863V (c.2588G > T)* ERBB2: V797M (c.2389G > A)*
264		PDGFRA: V824I (c.2470G > A)*
276	EGFR: P753L (c.2258C > T)	
MSI-H23		PDGFRA: L826P (c.2477T > C)
MSI-H24	FGFR3: A391V (c.1172C > T)	
MSI-H25		FGFR3: G370D (c.1109G > A)*
MSI-H29		ERBB4: T926M (c.2777C > T)* FGFR1: R148H (c.443G > A)

* Mutation at this amino acid position, but not this unique nucleotide mutation, is reported in COSMIC.

### Insertion/deletion mutations involving homopolymer or short tandem repeat regions

In MSS tumors, 27 of 34 (79%) indel mutations were detected in the *APC* gene as compared to 6 of 15 (40%) indel mutations in MSI-H tumors (data not shown) (*P* = 0.01). In MSI-H tumors, the most common indel mutations were detected in the *PTEN* gene (8/15 vs. 0/34 in MSS tumor, *P* < 0.001). Mutations occurred at homopolymer regions in 22 of 34 (65%) indel mutations of MSS tumors and in 13 of 15 (87%) indel mutations of MSI-H tumors (Table 5). All 8 *PTEN* indel mutations in MSI-H tumors involved a homopolymeric region including mono-, di-, and tetra-nucleotide repeats (Table 3). There were 3 p.K267fs mutations and 3 p.N323fs mutations with deletion of one adenine at a homopolymer region of 6 consecutive adenines. Overall, MSI-H tumors showed a significantly higher incidence of indel mutations involving homopolymer regions (0.45 vs. 0.19 per tumor), especially in the *PTEN* gene (0.28 vs. 0 per tumor, *P* < 0.001), but not in the *APC* gene (0.17 vs. 0.16 per tumor) (Table 5).

**Table 5:** Insertion/deletion (indel) mutations involving homopolymer regions

	Repeat region per indel*			Repeat region per tumor^†^		
	MSS	MSI-H	*P* value	MSS (*n* = 113)	MSI-H (*n* = 29)	*P* value
*APC*	18/27 (67%)	5/6 (83%)	NS	0.16	0.17	NS
*PIK3CA*	2/2 (100%)	0/0	NA	0.02	0	NS
*PTEN*	0/0	8/8 (100%)	NA	0	0.28	<0.001
*TP53*	2/4 (50%)	0/1	NA	0.02	0	NS
Others	0/1	0/0	NA	0	0	NS
Total	22/34 (65%)	13/15 (87%)	NS	0.19	0.45	<0.01

NA: not analyzed.

NS: not significant.

* Denominator indicates total number of indel mutations; numerator indicates number of indel mutations involving homopolymer regions.

† Indel mutations involving homopolymer regions per tumor.

## DISCUSSION

In this study, we compared mutations within 50 selected oncogenes and tumor suppressor genes in 113 MSS CRCs and 29 MSI-H CRCs using the AmpliSeq Cancer Hotspot Panel. MSI-H CRCs showed a significant higher mutation frequency than MSS CRCs. The difference was more remarkable when mutations within the 9 genes most commonly involved in the oncogenesis of CRCs were excluded from analysis. MSS CRCs showed higher incidences of mutations in the *APC*, *KRAS*, and *TP53* genes, while MSI-H CRCs showed higher incidences of mutations in the *BRAF*, *PIK3CA*, and *PTEN* genes as well as mutations in the RTK gene family. However, one would not be able to determine whether a specific tumor was MSS or MSI-H by simply looking at which genes were mutated.

Certain patterns of mutations in the AmpliSeq panel were associated with MSI-H; however, no criteria completely identified these tumors. Accordingly, CRCs with 6 or more mutations predicted MSI-H (Figure 1A, sensitivity = 0.14, specificity = 0.98). After removal of the 9 common genes, 2 or more mutations predicted MSI (Figure 1B, sensitivity = 0.45, specificity = 0.99). There was one parameter that differentiated the MSS tumors from the MSI-H tumors: more than 1 indel mutation in homopolymeric regions in any of the 29 MSI-H tumors. Of the 29 MSI-H tumors, 3 had 2 indel mutations in homopolymeric regions, but none of the MSS tumors had this feature (Figure 1C, sensitivity = 0.10, specificity = 1.00).

In the past, the MSI assay has been used to screen for Lynch syndrome according to the Amsterdam criteria or the revised Bethesda criteria [39]. The updated guideline for 2015 from the National Comprehensive Cancer Network suggests testing all CRC patients 70 years old or younger or meet revised Bethesda guidelines to screen for Lynch syndrome as well as patients with stage II CRCs to avoid 5-FU based adjuvant therapy [26, 40]. (available at http://www.NCCN.org) MSI status and *BRAF* mutation have also been combined for prognostic stratification of CRCs [41, 42]. Based on the recent paper by Le and colleagues, MSI status appears to be a predictive marker in the selection of patients who benefit from anti-PD-1 immunotherapy [25].

Defective MMR can be caused either by germline mutations of the MMR genes in Lynch syndrome or by somatic hypermethylation of the *MLH1* gene and rarely, somatic mutations of the MMR genes in sporadic CRCs [43, 44]. Defective MMR leads to hypermutation of the genome, possibly generating tumor neoantigens [14, 21, 45]. In retrospect, this may explain the seminal observation of the profound lymphocyte infiltration [18] and better outcomes in CRCs with MSI [20, 46, 7]. Recently, Le. *et al*. also reported that the presence of MSI-H non-CRC tumors (endometrial, ampullary or cholangiocarcinoma, small bowel, and stomach) predicts an impressive response rate in patients and that somatic mutation loads were associated with prolonged progression-free survival.^25^ Prediction of benefit from immunotherapy by mutational loads was also demonstrated in melanomas treated with ipilimumab or tremelimumab, antibodies against cytotoxic T-lymphocyte antigen 4 (CTLA-4), and in non-small cell lung cancers treated with pembrolizumab [22, 24]. These results suggest that the mutational load or neoantigen load itself may prove to be the best predictor in the selection of candidates for immunotherapy [23].

In this study, we confirmed a higher mutation frequency in MSI-H CRCs using the AmpliSeq Cancer Hotspot Panel. This relatively “small” 50-gene NGS panel also detected a large number of mutations in two metastases from a single patient (CRC 131). It is unclear whether the additional independent mutations occurred in the primary prior to metastasis, within the metastases following metastatic spread, or some combination of the two. The results suggest that this specimen is highly likely a consequence of a germline or somatic mutation in the *POLE*, *POLD1*, *MUTYH*, or other DNA replication/repair genes [16]. Mutations in the exonuclease domain of the DNA polymerase *POLE* and *POLD1* impair polymerase proofreading and lead to an extremely high rate of base substitution mutations [47]. Recently, *POLE* mutations were also detected in 7–12% endometrial cancers and were also associated with favorable prognosis [48]. *POLE* defect induced mutations can also elicit an anti-tumor response, suggesting that *POLE* mutations may be another predictive marker for response to anti-PD-1 or anti-PD-L1 immunotherapy [49, 25].

Our study suggests that a small NGS panel may be able to select tumors with hypermutation for anti-PD-1 or anti-PD-L1 immunotherapy. This approach may identify not only MSI-H tumors, but also MSS tumors with hypermutation. However, in the clinical diagnostics setting, specimens from selected patients often have a low tumor cellularity [28, 50]. In our retrospective quality assessment analysis of the NGS assay, *KRAS* mutations of less than 10% mutant alleles were observed in 5% of *KRAS*-mutated CRC specimens [31] and in 14% of *KRAS*-mutated lung cancer specimens (data not shown). An assay with a limit of detection of 5–20% tumor cellularity, such as the current MSI assay in this study, may not be able to detect MSI in tumors with extremely low cellularity, where IHC is probably a better tool. In addition, estimation of tumor cellularity itself is neither accurate nor precise, particularly in those with less than 10–30% tumor cellularity [51, 52]. Accordingly, a MSS finding with a tumor cellularity around or below the level of limit of detection may indeed be a false negative result. NGS assays used for mutational profiling have demonstrated a limit of detection of 2% or lower mutant allele [28, 30]. The detection of one or more initiating driver mutations can also be used to confirm the tumor cellularity estimated by pathologists. This is particularly helpful for pancreatic ductal adenocarcinomas, notoriously known for scattered tumor infiltration within the normal tissue.

Differential mutational profiling of CRCs with or without MSI has been reported in a large cohort of CRCs [16]. In the current study, we confirmed the higher *APC*, *KRAS* and *TP53* mutations in MSS tumors and the higher *BRAF* mutations in MSI-H tumors. Since *TP53* mutations often occurred later in the carcinogenesis, we compared *TP53* mutations in selected stage I-III patients. MSS CRCs still showed a higher *TP53* mutation frequency. We also confirmed a higher *PTEN* mutation frequency in MSI-H CRCs [53, 54]. In the current study, only 3 *PTEN* mutations were detected in 113 MSS CRCs (3%) as compared to 13 *PTEN* mutations detected in 8 of the 29 MSI-H CRCs (28%). The overall mutation frequency involving the mTOR pathway that includes the *PTEN*, *PIK3CA*, and *AKT1* genes was also significantly higher in MSI-H CRCs (55% vs. 18% in MSS CRCs). Further studies may be warranted in order to test if MSI-H tumors also respond to targeted therapy against the mTOR pathway.

Eight of the 13 *PTEN* mutations observed in MSI-H CRCs were indel mutations involving repeat regions and causing frameshifts. The two hot spots for indel mutation at the (A)_6_ repeats within exons 7 and 8 of the *PTEN* gene have been reported previously [53, 54]. This is not surprising given that mononucleotide runs of 6 bases are ∼1000 times more likely to undergo frameshift mutations than those of only 2 bases [15]. Other common genes with frameshift mutations in MSI-H CRCs include *TGF-BRII*, *TCF4*, *IGF2R*, *BAX*, *MSH6*, and *MSH3* [55, 39]. Indel mutations commonly induce truncated protein products with long stretches of foreign amino acid sequence (average ∼21 amino acids, based on 3 stop codons per 64 total codons) as potential neoepitopes to promote an anti-tumor immune response, in contrast to relatively subtle missense mutations caused by single base substitutions. Further studies may be needed to clarify if indel mutations involving repeat regions in MSI-H tumors contribute highly significantly to tumor responsiveness to anti-PD-1 or anti-PD-L1 immunotherapy and whether missense hypermutations in MSS *POLE*-mutated tumors may induce sufficient neoantigens for immunotherapy. It remains to be seen whether immunization with these tumor-specific neoepitopes might augment anti-PD1 therapy.

The AmpliSeq panel also detected a higher mutation frequency within the RTK families, including the *EGFR*, *KIT*, *PDGFRA*, *ERBB2*, *ERBB4*, *FGFR1* and *FGFR3* genes, in MSI-H CRCs. These may also provide additional potential targets for tyrosine inhibitors [56, 57, 58]. However, further studies are needed to elucidate if these mutations in fact drive the evolution of tumors. These results will need independent confirmation in future studies.

In this study, we confirmed a higher mutation frequency and a distinct mutational profiling in CRCs with MSI. NGS assays may be designed to detect driver mutations for targeted therapeutics and to identify hypermutations for anti-PD-1 and anti-PD-L1 immunotherapy. Further studies may be warranted to elucidate if MSI-H CRCs can benefit from targeted therapeutics against the mTOR pathway and to confirm if hypermutated MSS tumors may also respond to anti-PD-1 and anti-PD-L1 immunotherapy.

## MATERIALS AND METHODS

### Materials

Between April 2013 and November 2014, 310 formalin-fixed paraffin-embedded (FFPE) specimens with a diagnosis of colorectal cancer were submitted to the Molecular Diagnostics Laboratory at The Johns Hopkins Hospital for mutational profiling of the colorectal cancer panel as described previously [31]. Of these, 142 CRCs were also tested for MSI, and these samples are the focus of this retrospective study that consists of a mixture of primary and metastases. One hematoxylin & eosin (H & E) slide followed by 5–10 unstained slides and one additional H&E slide were prepared with PCR precautions. The H&E slide was examined and marked by a pathologist for subsequent macro-dissection of FFPE neoplastic tissues from 3–10 unstained slides of 5- or 10-micron thick sections. DNA was isolated from area(s) designated by the pathologist using the Pinpoint DNA Isolation System (Zymo Research, Irvine, CA), followed by further purification via the QIAamp Mini Kit (Qiagen, Valencia, CA) [32]. The Johns Hopkins Medicine institutional review board granted approval for this study.

### Next generation sequencing

NGS was conducted using the AmpliSeq Cancer Hotspot Panel (v2) for targeted multi-gene amplification, as described previously [30, 33]. Briefly, we used the Ion AmpliSeq Library Kit 2.0 for library preparation, the Ion PGM Template OT2 Kit and Ion OneTouch-2 Instrument for emulsion PCR and template preparation, and the Ion PGM 200 Sequencing Kit V2 with the Ion 318 V2 Chip and Personal Genome Machine as the sequencing platform (Life Technologies, Carlsbad, California). The DNA input was up to 30 ng, as measured by Qubit 2.0 Fluorometer (Life Technologies). Up to 7 patient specimens were barcoded using Ion Xpress Barcode Adapters (Life Technologies) for each Ion 318 V2 chip. At least one control (rotating between a non-template control, a normal peripheral blood control from a male, and/or positive control specimens) was included in each chip. The positive control was a mix of DNA from several cell lines with known mutations as described previously [31].

In our initial clinical validation of this NGS assay for mutation detection of the *KRAS*, *BRAF* and *EGFR* genes, 16 FFPE cancer-free specimens and 118 cancer specimens with known mutation status were included to validate the 6 traditional analytic performance characteristics recommended by the Next-Generation Sequencing: Standardization of Clinical Testing Working Group [34, 30]. We demonstrated that the NGS assay is sufficiently robust for detection of point mutations and short insertion/deletion mutations, with attention to potential artifacts, such as artifacts observed in the homopolymer regions as well as artifacts due to mispriming events intrinsic to multiplex PCR-based assays [35]. A background noise cutoff of 2% was chosen for single nucleotide variations based on analysis of 16 non-neoplastic FFPE tissues during the validation processes [30, 36]. The assay was also validated for clinical reporting of the *AKT*, *ERBB2*, *KIT*, *NRAS*, *PDGFRA* and *PIK3CA* genes and for the whole 50 gene panel (data not shown). All specimens submitted for the colorectal cancer panel were initially analyzed and reported for *BRAF*, *KRAS*, *NRAS* and *PIK3CA* genes as described previously [31]. Following de-identification and assignment of new case numbers to these 142 samples, mutations within the 207 amplicons of the whole 50 gene panel were retrospectively analyzed by the Torrent Variant Caller (Life Technologies) and confirmed by direct visual inspection of the binary sequence alignment/map file using the Broad Institute's Integrative Genomics Viewer [37].

The AmpliSeq hotspot panel is designed to detect the majority of reported mutations within genes, however it does not analyze every exon within each gene and so likely underestimates mutations within tumor suppressor genes, such as *APC* and *hMLH1*.

### Microsatellite instability test

Multiplex PCR amplification of 5 mononucleotide microsatellite loci (BAT-25, BAT-26, NR-21, NR-24, and MONO-27) and 2 pentanucleotide microsatellite loci (Penta C and Penta D) was performed as described previously using the MSI Analysis System (Promega Corp., Madison, WI), according to the manufacturer's instructions [10, 38]. Amplification products were analyzed by using the ABI 3130 × L capillary electrophoresis instrument (Applied Biosystems). The CRC is designated as MSI-high if novel allele lengths are identified in tumor tissues at 2 or more microsatellite loci, as compared with the normal/germline [13, 11].

### Statistical analysis

Chi-square test, Fisher exact test, or Student's *t*-test was performed to calculate *P* values as appropriate. The data were not corrected for multiple hypothesis testing.
